# Predicting Risk of Imported Disease with Demographics: Geospatial Analysis of Imported Malaria in Minnesota, 2010–2014

**DOI:** 10.4269/ajtmh.18-0357

**Published:** 2018-07-30

**Authors:** Elizabeth H. Lee, Robin H. Miller, Penny Masuoka, Elizabeth Schiffman, Danushka M. Wanduragala, Robert DeFraites, Stephen J. Dunlop, William M. Stauffer, Patrick W. Hickey

**Affiliations:** 1 The Uniformed Services University of the Health Sciences, Bethesda, Maryland;; 2 The Henry M Jackson Foundation, Bethesda, Maryland;; 3 Minnesota Department of Health, St. Paul, Minnesota;; 4 Hennepin County Medical Center, Minneapolis, Minnesota;; 5 University of Minnesota, Minneapolis, Minnesota

## Abstract

Although immigrants who visit friends and relatives (VFRs) account for most of the travel-acquired malaria cases in the United States, there is limited evidence on community-level risk factors and best practices for prevention appropriate for various VFR groups. Using 2010–2014 malaria case reports, sociodemographic census data, and health services data, we explored and mapped community-level characteristics to understand who is at risk and where imported malaria infections occur in Minnesota. We examined associations with malaria incidence using Poisson and negative binomial regression. Overall, mean incidence was 0.4 cases per 1,000 sub-Saharan African (SSA)–born in communities reporting malaria, with cases concentrated in two areas of Minneapolis–St. Paul. We found moderate and positive associations between imported malaria and counts of SSA- and Asian-born populations, respectively. Our findings may inform future studies to understand the knowledge, attitudes, and practices of VFR travelers and facilitate and focus intervention strategies to reduce imported malaria in the United States.

## INTRODUCTION

Malaria remains an important health concern among U.S. travelers, particularly within sub-Saharan African (SSA) immigrant and refugee communities who make up the highest burden proportion of travelers visiting friends and relatives (VFR).^1^ Strategies to reduce the disease burden in this population have previously centered on identifying barriers to preventive care,^2–5^ culturally sensitive risk communication,^2,6^ as well as medical provider knowledge and practice.^7–9^ Furthermore, malaria prevention strategies and interventions have been more broad-based in the scope of information provided and intended audience rather than tailored to high-risk populations such as VFRs.^10^ Existing interventions have largely taken a top-down approach without solid understanding of knowledge, attitudes, and practices (KAP) of groups at greatest risk. Furthermore, there are limited data on the effectiveness of existing prevention strategies at the population level. This study reflects initial results from one line of effort within a larger multisite and multidisciplinary prevention study funded by the Centers for Disease Control and Prevention.

Minnesota has a large, diverse immigrant and refugee population and ranks in the top 10 states for absolute number of malaria cases.^11,12^ A tradition of robust health engagement in refugee and immigrant populations makes Minnesota an ideal state for assessing the impact of malaria interventions in high-risk communities (http://www.health.state.mn.us/divs/idepc/refugee/). An important first step toward addressing gaps in prevention for VFRs, this study leverages disease surveillance and open source census data to develop a model that can be adapted by researchers or health departments to other contexts to anticipate risk, target prevention efforts, and measure impact.^13^

## MATERIALS AND METHODS

### Study population.

Our cross-sectional analysis covered the entire state of Minnesota, which had an estimated population of 5,519,952 overall in 2016.^14^ In 2016, Minnesota reported a malaria incidence of 1.2 cases per 100,000 population, or a total of 66 cases.^15^ Annual malaria cases are distributed throughout the state, with highest annual burdens generally concentrated in urban areas.^15^ About three-fourths of the Minnesotan population resides in urban areas, with the remainder spread across large and small towns as well as rural areas.^16^ The state is known for having a large African diaspora (estimated 107,880 African-born in 2016),^14^ many of whom reside in the capital region of Minneapolis–St. Paul.^17^ Median household income in Minnesota in 2016 was 63,217 USD, and 6.9% of families were living below the poverty line.^14^

### Data sources and measures.

We extracted data for Minnesota from three sources: the American Community Survey (ACS), Department of Health routine malaria surveillance data, and physician and pharmacy counts. The U.S. Census Bureau annually collects and publicly releases demographic and socioeconomic ACS data for individuals and households (https://www.census.gov/geo/maps-data/data/tiger-data.html). To protect participant privacy, ACS data which are representative at smaller geographies are released in either in 5-year period estimates or as a 5% resample of individuals from the nationally representative 5-year aggregate sample.^18^ We analyzed the 2010–2014 Minnesota subset of ACS files available in median 5-year summary estimates for ZIP Code Tabulation Areas (ZCTAs), which aggregate annually collected data covering 60 months. ZIP Code Tabulation Areas are generalized areal representations of U.S. Postal Service ZIP Codes which are geographically defined by the U.S. Census Bureau. We selected ZCTAs as the geographic unit of analysis based on availability of all variables considered for analysis, as well as for practical considerations to readily inform related, ongoing community-based activities led by the research team in the Minneapolis–St. Paul region. ZIP Code Tabulation Areas were eligible for inclusion if they fell fully within the state of Minnesota and had a population of one or more persons.

Malaria case information was derived from de-identified malaria surveillance data for 2010–2014 provided by the Minnesota Department of Health and included case year, patient ZIP Code of residence at the time of diagnosis, age, gender, country of birth, country of travel, and country of residence before most recent travel. Cases were eligible for inclusion if a ZIP Code was provided in the record. We aggregated cases to the ZIP Code level.

Addresses for Minnesota physicians and pharmacies were obtained through SK&A, QuintilesIMS company (Danbury, CT) (www.skainfo.com), for the year 2016. The only physician practices included were limited to those within 10 medical specialties we considered to be most likely to provide pre/post-travel care: adolescent medicine, emergency medicine, family practitioner, general practitioner, infectious disease specialist, internal medicine/pediatrics, internist, pediatrician, preventive medicine specialist, and urgent care specialist. Physician and pharmacy data were aggregated to the ZIP Code level.

The primary independent variable for this analysis was the median count of population reporting place of birth in an SSA country endemic for malaria. Sociodemographic variables selected a priori and based on literature review included total (median) population count, median household income in 2013-adjusted U.S. dollars for ZCTAs, and median counts of the following subpopulations: those aged 25 years or older with at least a 12th grade equivalent education, those with any health insurance, and those who speak a language other than English at home.^2,7,11,19^ We also considered median count of persons reporting Asian birth, where Asia is the second greatest region of acquisition (11% of reported U.S. malaria cases in 2013).^20^ Our primary outcome was count of malaria cases diagnosed in 2010–2014 for Minnesota ZCTAs. Variables were measured as median period estimates, in keeping with the form provided in the ACS datasets for the 5-year summary estimates. We used counts rather than proportions as proportions proved to be unstable because of small sample sizes.

### Data analysis.

**Figure 1.**
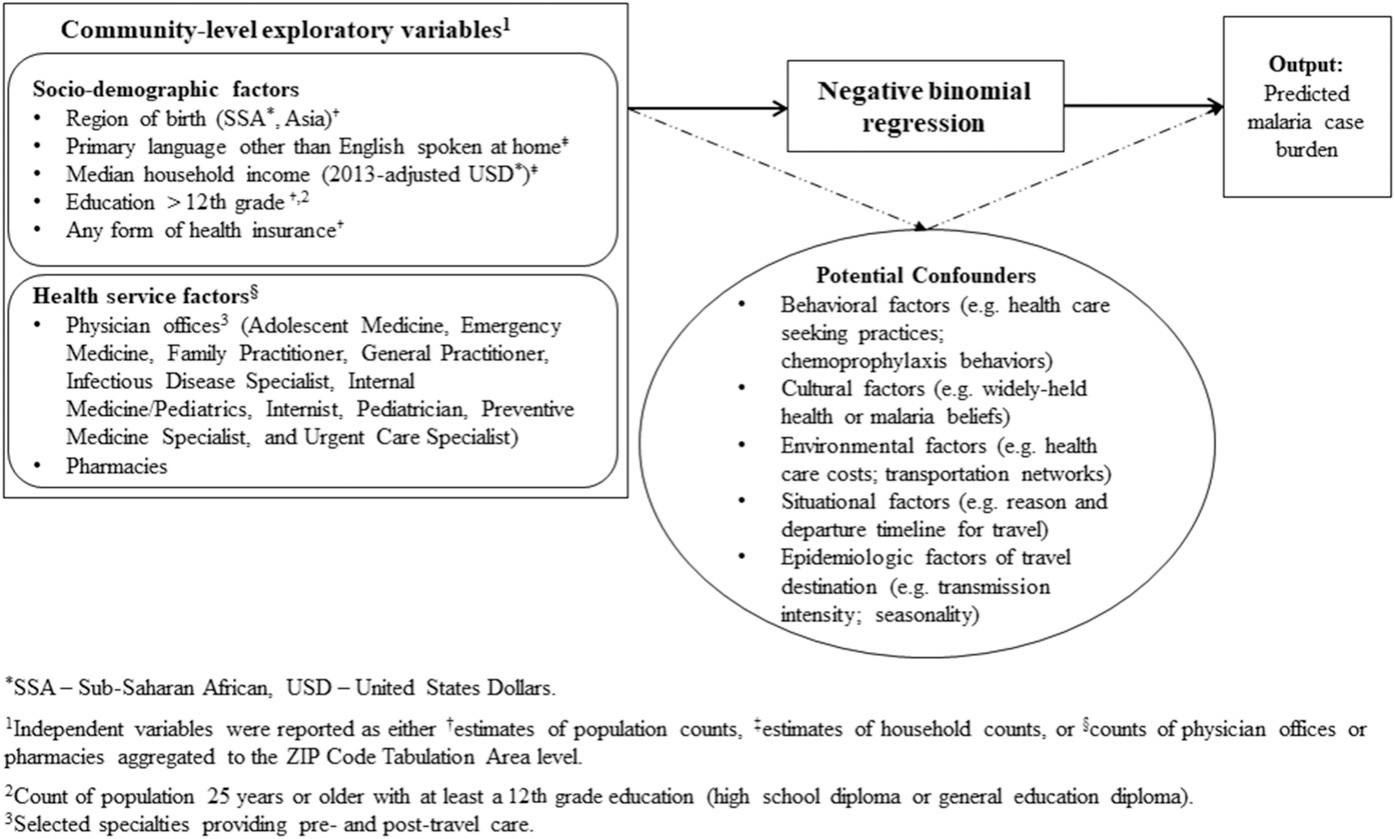
Analytic framework for the predicting risk of imported disease with demographics (PRIDD) method. This framework depicts the analytic approach for exploring the relationship between community-level independent sociodemographic and health service factors with malaria case burden. The potential for confounding of this relationship by other behavioral, cultural, environmental, and situational factors is indicated by the broken line to the oval component demonstrating examples of potential confounders which are not controlled for using the PRIDD method with American Community Survey data because of availability of variables in used datasets.

We developed a method for predicting risk of imported disease with demographics (PRIDD), or the PRIDD method (Figure 1). We calculated median counts and frequencies for characteristics of malaria cases and descriptively mapped and examined the geographic distribution of sociodemographic characteristics and malaria case counts at the community or ZCTA level in a geographic information system (GIS). Malaria incidence in the SSA-born population was calculated as malaria case totals divided by the median SSA-born population for the 2010–2014 period. The primary independent variable for this analysis, the count of population reporting place of birth in an SSA country endemic for malaria, was selected in lieu of a similar variable, SSA ancestry, after examining respective Spearman’s rank correlation coefficient strengths with malaria case counts. Unadjusted and adjusted imported malaria risk estimates for ZCTAs were calculated using negative binomial regression with robust variance estimation and case-wise deletion. Population was included as an offset in the final model, to provide risk estimates per 1,000 population. We performed descriptive analyses, including geospatial mapping in ArcGIS 10.3 (ESRI, Redlands, CA), GraphPad Prism v.6 (GraphPad Software, La Jolla, CA), and SPSS v.22 (IBM, Armonk, NY) and conducted exploratory regression analyses in Stata 14.0 (StataCorp., College Station, TX).

### Ethical approval.

The study protocol was reviewed and deemed for nonhuman subjects research by the institutional review boards of the Uniformed Services University of the Health Sciences, Bethesda, MD; the Minnesota Department of Health, St. Paul, MN; and the University of Minnesota, Minneapolis, MN, because all data were de-identified.

## RESULTS

Of 891 total Minnesota ZCTAs, 879 were eligible for inclusion in our analytic sample. We excluded five ZCTAs which had no population and seven (< 1%) ZCTAs, each with no reported malaria cases, which were missing data for median household income. Comparison of the medians of sociodemographic and health service characteristics suggested ZCTAs with missing data did not differ from ZCTAs in the analytic sample which reported no cases. We matched all (100%) physicians and pharmacies by ZIP Code to ZCTAs, and 267 (98%) of eligible malaria cases were matched to ACS ZCTA data in shapefile format in a GIS. Excluded cases included five persons missing ZIP Code of residence and seven persons who resided in other states but had received their diagnosis in Minnesota. Minnesota malaria surveillance reports are officially based on location of diagnosis and also include ZIP Code of residence. We used the latter to construct case counts.

### Descriptive characteristics of Minnesota malaria cases.

Between 2010 and 2014, the Minnesota Department of Health reported 272 malaria cases across 101 (10%) ZCTAs, primarily but not exclusively concentrated in metropolitan areas. Fifty-nine percent (161) of patients were male, and 49% of cases (133) occurred in individuals aged 18–44 years (Table 1). West Africa, East Africa, Central Africa, and Southern Asia were the most commonly reported regions of travel with 56.6% (154), 18% (49), 6.3% (17), and 6.4% (17) of patients, respectively (Supplemental Figure 1). One hundred and fifty-six patients (57%) reported being born outside the United States, suggesting many of the patients may have been VFRs. Country of birth was unknown for an additional 32% (86) of patients.

**Table 1:** Summary of malaria cases reported to the Minnesota Department of Health from 2010 to 2014

Malaria patient characteristics		No. patients (%)
Case year	2010	50 (18.4)
	2011	47 (17.3)
	2012	58 (21.3)
	2013	67 (24.6)
	2014	50 (18.4)
	Total	272 (100)
Gender	Male	161 (59.2)
	Female	111 (40.8)
Age range	Under 5 years	15 (5.5)
	5–17 years	34 (12.5)
	18–44 years	133 (48.9)
	45–64 years	78 (28.7)
	65 years and older	12 (4.4)

### Distribution of malaria cases and sociodemographic characteristics at the community level.

**Figure 2.**
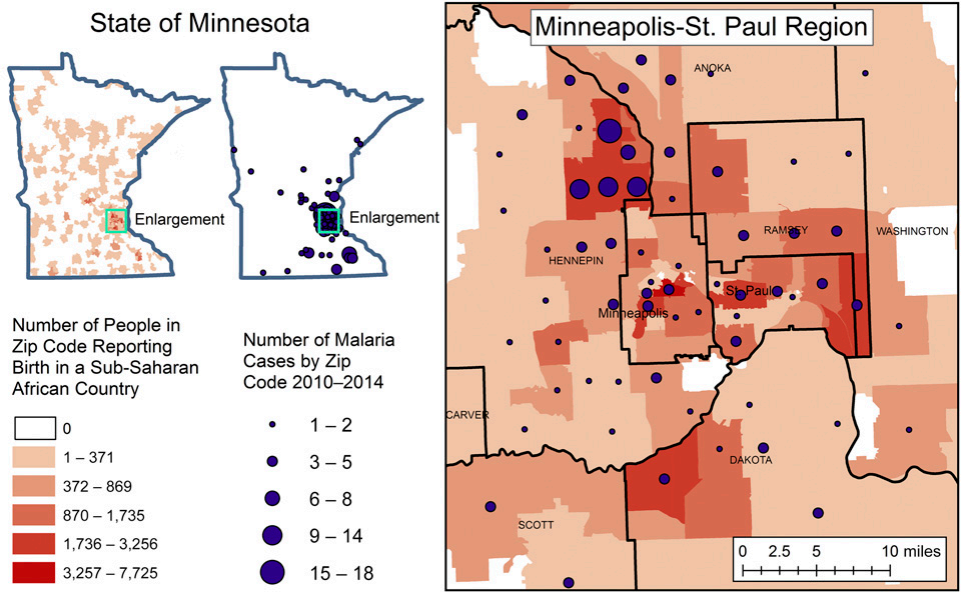
Imported malaria cases (blue dots) overlaid on the number of people reporting birth in a sub-Saharan African country by ZIP Code Tabulation Area (ZCTA) (red area symbols). Malaria cases cluster with ZCTAs reporting larger populations of individuals reporting birth in sub-Saharan African countries. Malaria case Zip Codes were obtained from Minnesota Department of Health. Location of birth was obtained from the American Community Survey.

**Figure 3.**
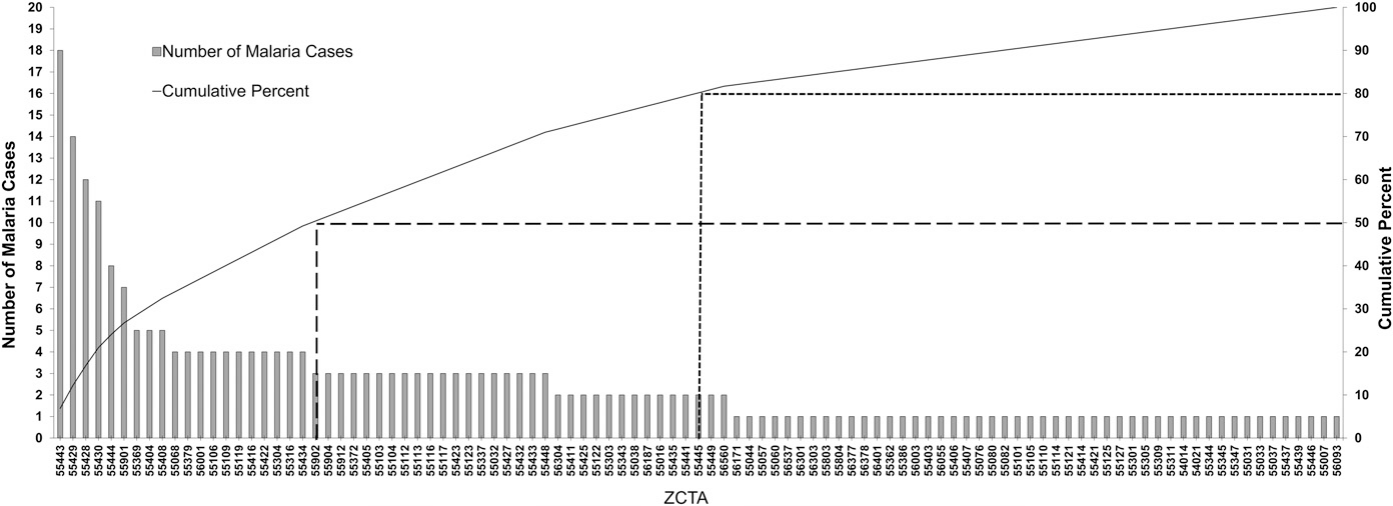
Plot of number of imported malaria cases by Zip Code. Imported malaria cases are concentrated in select Minnesota ZIP Code Tabulation Areas (ZCTAs). Of the 101 MN ZCTAs reporting malaria cases from 2010 to 2014, 51 ZCTAs reported 80% of imported malaria cases in Minnesota from 2010 to 2014 (dotted line). Twenty ZCTAs reported 50% of imported malaria cases in Minnesota from 2010 to 2014 (dashed line).

We then looked at the distribution of SSA- and Asian-born populations in Minnesota, which were found to be concentrated in several metropolitan areas, including the Minneapolis–St. Paul metropolitan region (Figure 2, Supplemental Figure 2). Similarly, results of ZCTAs ranked by malaria case count indicated that cases were concentrated geographically, with 20 ZCTAs contributing to 50% of cases and 51 ZCTAs contributing to 80% (Figure 3). Mapped cases overlaid on SSA-born population confirmed a concentration of both in metropolitan Minneapolis–St. Paul ZCTAs (Figure 2).

**Figure 4.**
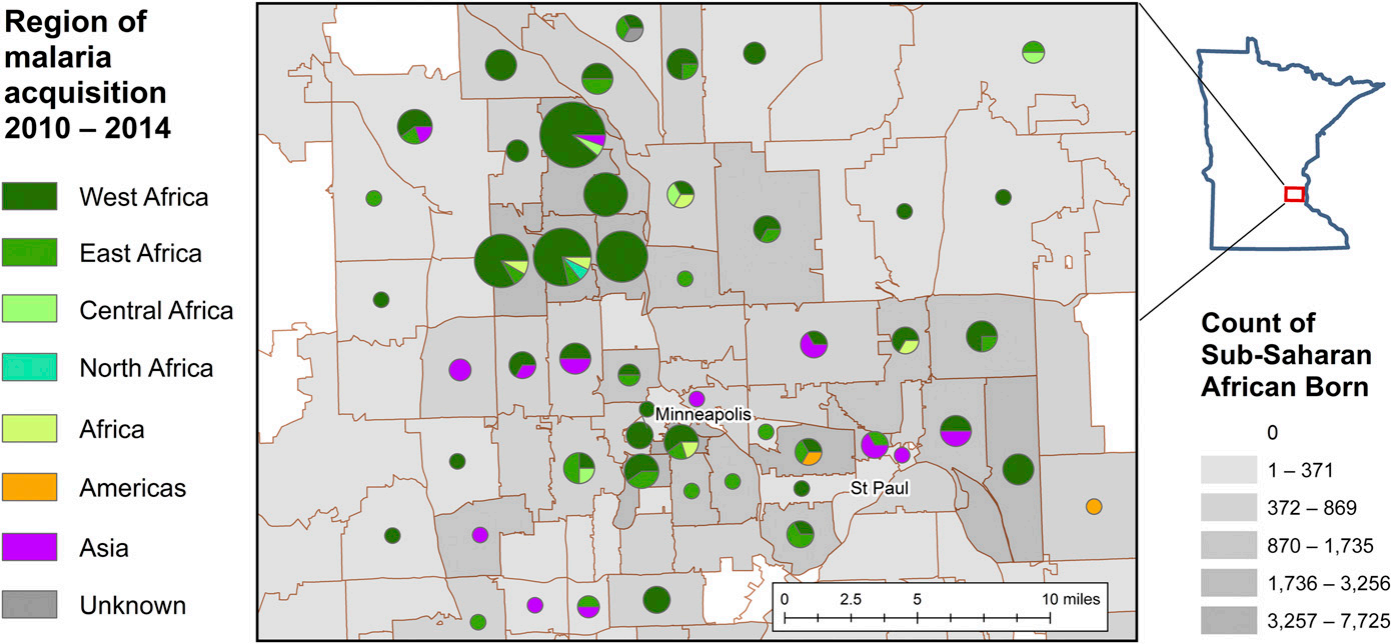
Pie charts of imported malaria cases by region of travel/acquisition overlaid on the number of people reporting birth in a sub-Saharan African country by ZIP Code Tabulation Area (ZCTA). Regions of malaria case acquisition match ZCTAs reporting large populations of individuals reporting birth in sub-Saharan African countries. The size of the pie chart circle is based on the number of malaria cases in the ZCTA. The largest circle represents 18 malaria cases, the smallest circle represents one malaria case.

Focused analysis of the Minneapolis–St. Paul region suggested that malaria cases were found in ZCTAs with higher populations of individuals reporting birth in SSA and Asian countries (Supplemental Figure 2). Moreover, patients who reported acquisition of malaria in sub-Saharan Africa tended to reside in ZCTAs with large SSA-born populations (Figure 4). ZIP Code Tabulation Areas with larger malaria burdens for the 2010–2014 period also tended to have larger populations of persons speaking a primary language other than English at home, lower median household income, more insured households, and fewer individuals with at least a 12th grade education (Supplemental Figure 2). Finally, we identified a subset of ZCTAs with the greatest malaria case burden in Hennepin County, which also had fewer pharmacies and physicians per 1,000 population compared with the state average.

### Community risk factors for imported malaria.

We explored potential unadjusted and adjusted community or ZCTA-level risk factors for malaria (Table 2). Unadjusted results suggested possible associations with every variable of interest. Results of Spearman’s rank correlation testing for case counts with ancestry and foreign-birth variables indicated that SSA birth was most strongly correlated (*r* = 0.5566, *P* < 0.001); thus, we chose to use SSA birth over ancestry in further population analyses. Exploratory results of our adjusted model with a population offset suggested four modest significant predictors of malaria risk per person in the population. Sub-Saharan African birth, Asian birth, and higher level of education were positively associated, whereas the language other than English spoken at home was protective against increased malaria risk.

**Table 2:** ZIP Code Tabulation Area–level factors associated with unadjusted and adjusted risk of imported malaria per 1,000 population

Variable	Unadjusted risk*	95% CI	Adjusted risk*†	95% CI
Median count of population reporting single ancestry as sub-Saharan African	0.9	(0.7, 1.2)	–	–
Median count of sub-Saharan African-born	1.2	(0.9, 1.5)	1.2	(0.8, 1.6)
Median count of Asian-born	0.8	(0.5, 1.2)	0.4	(0.1, 0.7)
Median count of all foreign-born	0.4	(0.3, 0.5)	–	–
Median household income in 2013 inflation-adjusted USD (per 1,000 USD)	0.7	(−9.2, 10.6)	–	–
Education of at least a GED or high school diploma for population of 25 years or older	0.1	(0.1, 0.1)	> 0.0	(> 0.0, 0.1)
Count of physicians	19.5	(7.8, 31.3)	–	–
Count of pharmacies	135.0	(70.7, 203.2)	–	–
Median count of population with any health insurance	0.6	(0.4, 0.7)	–	–
Median count of population reporting a primary language spoken at home other than English	0.3	(0.2, 0.4)	−0.2	(−0.3, < 0.0)

CI = confidence interval; GED = general education diploma; USD = United States dollars. Modeled using negative binomial regression and robust variance estimation.

* Unadjusted risk and adjusted risk are calculated as number of expected malaria cases per 1,000 population, except for median household income which is calculated as risk per 1,000 households per 1,000 USD.

† Adjusted for total population, estimated median population reporting sub-Saharan African birth, estimated median population reporting Asian birth, estimated median population that reports speaking a primary language other than English at home, and estimated median population above age 25 with at least a high school diploma or GED. Akaike’s information criterion: 603.014.

## DISCUSSION

**Figure 5.**
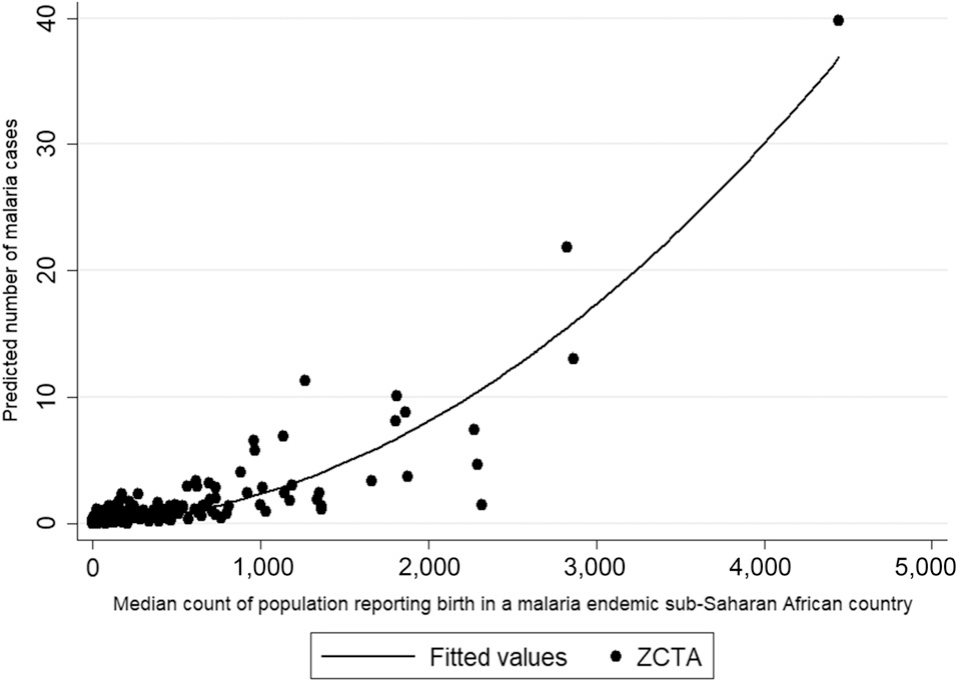
Scatterplot of sub-Saharan African-born median counts based on 2010–2014 American Community Survey data with predicted malaria cases. Sub-Saharan African birth and predicted number of malaria cases are positively associated.

Overall, our findings suggested a positive association between SSA-born population and malaria case burden for Minnesota ZCTAs concentrated in urban, metropolitan areas of Minneapolis–St. Paul (Figure 5), which is consistent with known epidemiology of the region. Comprising seven counties with a population estimated at 3,005,419,^21^ the Twin Cities region of Minneapolis–St. Paul has a large foreign-born population, with approximately 29% (69,400) of black residents and 62% (123,300) of Asian residents born overseas.^22,23^ Of foreign-born black and Asian residents, 57% and 65%, respectively, had resided in the United States for 10 or more years.^22,23^

Our results can be used to measure and longitudinally follow a geographically defined and denominator-based burden of disease and to identify associated demographic factors that can be used to assess risk at the population level. We calculated a cumulative incidence of 0.4 cases per 1,000 SSA-born population in ZCTAs reporting malaria for 2010–2014 for the entire state of Minnesota. Unadjusted findings indicated sociodemographic and health service access factors such as health insurance status and median household income were positively associated with imported malaria. After adjustment, four significant associations remained: education level, SSA-born population, Asian-born population, and the foreign language spoken at home. Sub-Saharan African-born population was strongly associated with case burden for 2010–2014, contributing an estimated 1.2 cases per 1,000 population. Interestingly, primary language other than English was negatively associated with malaria burden after adjustment. This may reflect newly established immigrant communities from non–English-speaking, malaria-endemic nations which have yet to fully assimilate or travel internationally or may represent populations from non–malaria-endemic or low-prevalence regions. The effect of this variable will be further studied in ongoing work in other geographic locales.

Our findings support existing evidence which suggests that imported malaria cases tend to occur in VFR travelers to sub-Saharan Africa or Asia and in persons of higher education.^7,8,11,24–29^ Although median household income appeared to increase as malaria cases increased in the unadjusted analysis, this characteristic was not significant in the adjusted model. By contrast, a similar case–control study in Canada found that for the adjusted relationship, as cases increased, median income decreased.^28^ U.S. and Canadian VFR populations could differ in this characteristic; in general, Minnesota immigrant communities tend to be older and well established^11^ and, thus, may have amassed wealth before first VFR travel. Alternatively, it is conceivable that this relationship follows a bell curve, with those at the upper and lower percentiles less at risk for malaria due to improved health care and education access or lowered ability to travel, respectively, with those in the middle at comparatively increased risk.

Models to predict infectious disease prevalence and dynamics use a variety of risk factors and methods, including confirmed cases, vector prevalence, reservoir populations, socioeconomic factors, host immunity, pathogen transmissibility, environmental conditions, climatic factors, and human population density.^28,30–32^ These models can be useful tools for epidemic surveillance, predicting disease emergence, and controlling disease outbreaks.^33,34^ Although malaria GIS and spatial modeling techniques are widely described in the literature,^35–41^ we adapt these to the field of travel medicine and imported malaria in the domestic U.S. setting which is novel. Our approach to modeling, PRIDD or the PRIDD method, is unique and practical, given malaria is imported rather than endemic to the United States and, thus, is heavily reliant on sociodemographic characteristics, population density, and confirmed case data to identify at-risk areas for intervention.

To date, community-level studies that identify specific groups at increased risk for imported malaria have been limited.^9^ Most studies have focused on KAP, or barriers to pretravel care-seeking using individual-level data.^2,3,42–44^ A prior study of imported pediatric malaria in the Washington, DC area demonstrated a mapping method for exploring the relationship of case distribution and density in relation to specific ethnic groups at the ZCTA level.^10^ A related study showed that access to antimalarial medications at local pharmacies varied across communities and was not consistently correlated with risk.^45^ Taken together, these studies demonstrate that defined communities of risk exist both demographically as well geographically, and helped inspire the present work. This insight highlighted the need for more efficient application of prevention resources that could target both at-risk individuals and the medical providers who serve their communities. By having access to reliable population estimates, especially if complemented by community-based surveys, this approach could mitigate the lack of denominator data in many retrospective analyses of malaria risk factors. This method could also be used to ensure that diagnostic and therapeutic resources are available to communities in greatest need. We enhanced the previous approach by mapping and extracting similar data for Minnesota in a GIS, which were then used to predict locations of communities at risk for malaria. By replicating the approach in multiple communities, a more robust and valid model will be developed. A similar concept was used in a Canadian case–control study to examine factors associated with imported malaria at the neighborhood level.^28^ Our method modeled risk using count data which is more suitable for estimating associations when prevalence of the outcome is greater than 10%.^46^ Other geographical approaches to studying imported malaria include passive surveillance, parasite speciation to identify likely region of acquisition, and use of airline travel data,^47–49^ but limitations on data availability due to privacy and proprietary concerns diminish the potential for wide use.^50^

This study had several limitations, which were partially data driven. As ACS data were self-reported, it is possible that respondent bias may have been introduced if certain factors such as median household income were under- or over-reported.^51^ However, we expect the effect of this misclassification in either direction to be minimal, particularly as data were aggregated to the community or ZCTA level.^51^ We did not have consistent data on reason for travel or on reported ethnicity of cases, which would have been helpful for case identification in non-VFR travelers. Consequently, our estimate of incidence in the SSA population may be biased upward. We were also missing some data for certain characteristics of the malaria case persons, such as region of birth (32%) and/or region of travel or malaria acquisition (< 1%). However, our sensitivity analysis of cases with unknown country of birth suggested that region of acquisition was largely an African or South/Southeast Asian malaria-endemic country 99% of the time; only 1% had an unknown region of acquisition, and country of birth composition for both groups was qualitatively similar. Consequently, it seems reasonable to hypothesize that many of these cases were VFR travelers returning to the same endemic country of their birth.

Furthermore, although we used the best available physician and pharmacy census datasets as well as state-reported malaria surveillance data, it is impossible to confirm the completeness of these datasets. For example, there may be unreported cases of malaria due to self-treatment or symptom mildness. If present and if systematically associated with community-level characteristics, these missing cases might partially explain why ZCTAs had low cases where high burdens were predicted.

Given results of correlation strength testing, we opted to use the variable for place of birth instead of total SSA ancestry as our primary exposure. In each case, misclassification might have been present, making either variable an imperfect proxy for the SSA community at risk for imported malaria. We calculated total population reporting any ancestry as a malaria-endemic country in SSA, excluding Cape Verde. We calculated the total foreign-born in SSA following United Nations geographical regions (http://millenniumindicators.un.org/unsd/methods/m49/m49regin.htm-africa), with the addition of Sudan (including South Sudan), and excluding Cape Verde. Ancestry count is likely to overestimate the population at risk by including anyone of SSA descent even if there are no longer familial ties to SSA, whereas foreign birth count excludes first generation descendants of immigrants who may be nonimmune children at increased risk for contracting malaria as VFRs. Using correlation results and this information, we opted to use foreign birth as the primary exposure of interest, which may have biased our results toward the null, yet by our estimation was a more conservative association to examine.

Although novel in its approach, our analysis was somewhat limited by the geographic unit of analysis and our inability to disaggregate data. It was not feasible to link individual cases from surveillance data to individual respondents in the ACS because of the survey sampling method to ensure, among other considerations, participation privacy. Instead, we aggregated cases to the ZIP Code level and linked ZIP Codes to ZCTA-aggregated publicly available census and commercial physician and pharmacy data. The effects of this were 4-fold. First, we were limited to variables captured in the ACS, which does not include potentially important factors such as duration of residence in the United States. Second, ZCTA-level variables cannot be further subset or disaggregated. For example, we could not examine SSA country of birth by age distributions for ZCTAs, which would allow for more nuanced characterization of populations. Third, by using ZCTAs as our unit of analysis, we were limited in our use of the data. Although we could descriptively explore surveillance data and aggregate cases to the ZCTA level, it was not feasible to consider specific case attributes such as age or gender in the adjusted model. Fourth, ZCTAs may be heterogeneous in population size making direct comparisons challenging, whereas other sampling units, for example, census tracts, tend to afford improved statistical uniformity.^52^ However, we attempted to mitigate this by using population as an offset in the adjusted model. Furthermore, we used ZCTAs for practical reasons, given ubiquitous familiarity with ZIP Codes and the intent of this work to readily inform ongoing community-based operations.

Notably, our calculation of cumulative malaria incidence in SSA-born populations in Minnesota was also limited by our inability to disaggregate ZCTA-level data and so is our best, albeit an imperfect, estimate. The denominator includes those on prophylaxis as there was no way to remove this group from reported ZCTA population estimates. Prophylaxis use might increase as immigrant populations become more established and financially secure, thereby resulting in a lower true cumulative incidence. Conversely, the influx of new immigrants who might be less inclined to take prophylaxis could offset the impact of increased prophylaxis use over time, although they might still retain improved acquired immunity.

Our study is the first to model and predict communities at risk for imported malaria using linked open source census and routine surveillance data or the PRIDD method. American Community Survey data are readily accessible and provide a potentially low cost alternative to primary data collection. These data are representative at the ZCTA level, increasing potential generalizability to communities with similar sociodemographic characteristics. We can adapt and apply the PRIDD method in other similar geographic municipalities to predict and validate where communities with increased risk for imported malaria may be present, and track trends over time. For example, this pilot project is part of a broader multisite imported malaria study funded by the U.S. Centers for Disease Prevention and Control, which will predict malaria risk in other United States communities. This may best apply to urban municipalities and those with known relatively large populations of SSA or Asian immigrants and may require model adjustments based on local context. Alternatively, it could support prevention efforts in newly established and growing immigrant communities so that public health needs and funding can be prioritized. In addition, future modeling work might explore whether the use of census tracts or blocks would improve model predictive performance and thus generalizability of findings.

Our methods may also be used to identify communities where the malaria burden has consistently been low, suggesting positive deviance from expected risk and providing an opportunity to study contributing factors. For example, a sensitivity analysis comparing reported versus predicted cases indicated that our model performs well overall but that in several instances reported malaria cases either positively or negatively deviated from predicted values (Supplemental Figures 3 and 4). In these situations, low reported cases were predicted to be high or vice versa. This information could be used to inform targeted pilots or expansion of existing travel medicine initiatives and longitudinally measure the effectiveness of prevention interventions. Furthermore, the PRIDD method could be applied to address similar challenges with other reportable illnesses, for example, Zika virus and typhoid fever.

The PRIDD method also demonstrates the use of mapping in a GIS to visualize and explore a less-understood issue of domestic health importance. With maps we determined the location of communities at risk to identify where target community engagement and interventions are necessary, the location of community assets which might be leveraged as part of an informed intervention response, and where future imported malaria cases are expected, given community characteristics. Geographic information system mapping is a powerful tool to visualize important spatial or temporal relationships that can help identify communities at risk and inform derived interventions.^34^

Furthermore, translation of findings from research to practice can aid practitioners in designing interventions for pretravel malaria prevention and counseling in at-risk populations.^6^ For example, study findings have informed our engagement of at-risk Minnesota communities through colocation of a community advisory board, focus groups held to learn about malaria KAP in these communities, and solicitation of community suggestions for acceptable interventions. This approach to public health may lead to more effective, lower cost interventions suitable for imported malaria—a relatively uncommon and focal illness—although targeting only those high-risk communities most in need of intervention.

Our work also highlights the need for continued engagement in SSA and Asian communities in terms of pretravel preventive interventions for malaria, particularly in the Minneapolis–St. Paul, Minnesota metropolitan region. West and East African as well as South Asian diasporas may benefit from concerted social and behavioral change communication (SBCC) and direct efforts to increase the uptake of travel medicine counsel before departure. Study findings can help tailor interventions with the goal of reducing malaria burden in these populations, particularly with respect to where at-risk communities are located. For example, we identified several high-risk communities northwest of Minneapolis through mapping of cases, low density of physicians expected to offer travel medicine services, and low pharmacy density relative to state averages of 0.41 physicians per 1,000 population and 0.13 pharmacies per 1,000 population (Supplemental Figure 2). In addition to SBCC approaches, these findings suggest strategies to address service accessibility may be important for increasing pretravel intervention uptake, and the need to collect further accessibility data on public transport networks and private automobile reliance.

The combination of existing census and surveillance data and their exploration in a GIS provides powerful analytics for determining where communities at high risk for imported malaria are located in the United States. VFR travelers continue to be at increased risk for diagnosis of malaria on return to the United States from malaria-endemic areas. Our findings can be used to geographically target suitable interventions. The PRIDD method may be applied to other geographic regions or the entire United States to help inform targeted interventions for reduction of the national burden of imported malaria.

## Supplementary Material

Supplemental figures
