# Supplementary material for: Predicting Risk of Imported Disease with Demographics: Geospatial Analysis of Imported Malaria in Minnesota, 2010–2014
Source: Am J Trop Med Hyg. 2018 Jul 30;99(4):978–86. doi: 10.4269/ajtmh.18-0357 (PMC6159573; doi:10.4269/ajtmh.18-0357)
Supplement: Supplementary file 1 [file tpmd180357.SD1.pdf]

Supplemental Figure 1. Imported malaria cases in Minnesota primarily reported in patients traveling to West Africa and of West African birth. A) Area of birth: 100 (37%) of 272 total malaria cases were in individuals reporting West African birth. B) Destination of malaria risk travel: The majority of imported malaria cases were acquired in West Africa (154 cases or 57%) and East Africa (49 cases or 18%). Regions are categorized based on the United Nations Geographical Regions (United Nations Statistics Division 2016).

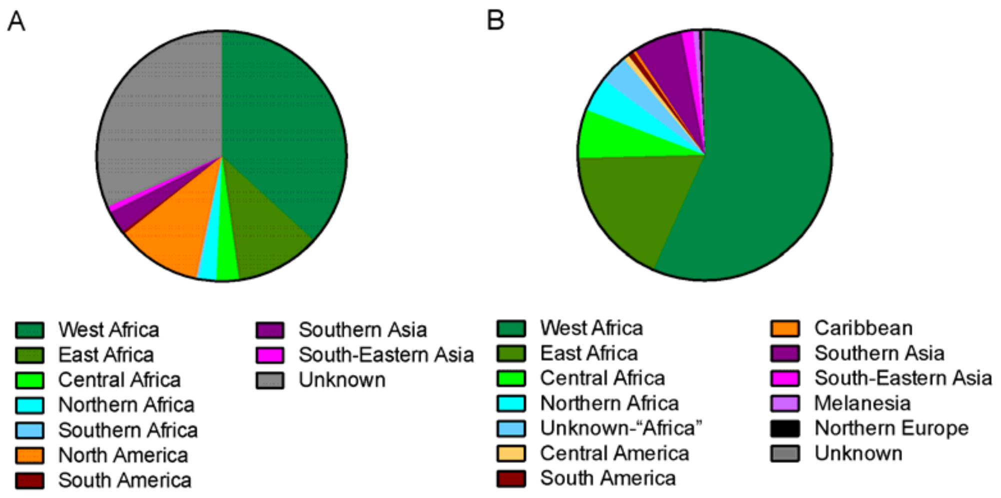

Supplemental Figure 2. Sociodemographic characteristics and selected physician and pharmacy densities mapped at the ZCTA-level for the Minneapolis-St. Paul region for 2010-2014. Spatial comparisons between locations of reported malaria cases and sociodemographic characteristics from ACS data include the number of people who reported birth in a sub-Saharan African country (A), the number of people who reported birth in an Asian country (B), the density of people who reported speaking a primary language other than English at home per 1000 population (C), the density of people who reported an education level of 12th grade/GED or above per 1000 population (D), the density of people who reported having health insurance per 1000 population (E), and median household income adjusted for 2013 United States Dollars (F). Comparison of the location of ZCTA-aggregated malaria cases and the density of: pharmacies per 1000 population (G) and selected physicians per 1000 population (H, see data and methods section for selected physician specialties) are also shown. ZCTAs within ten percent of the Minnesota state averages for pharmacy (G) and physician (H) densities are shown in yellow, ZCTAs with densities above the state averages are shown in light green and dark green, and ZCTAs below the state averages are shown in orange and red.

A

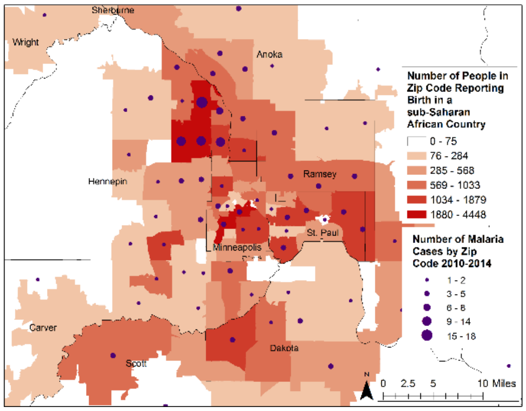

B

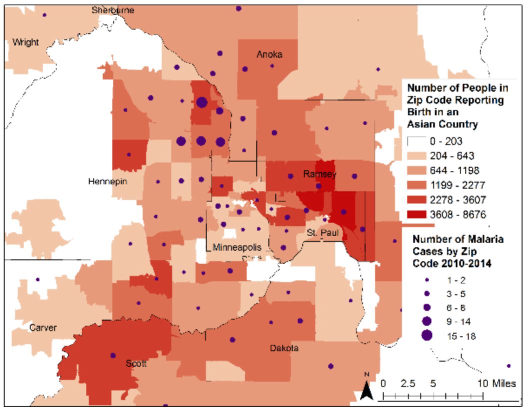

C

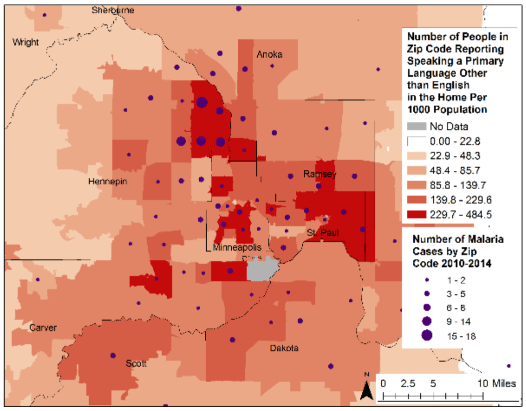
Sherburne
Wright
Anoka
Hennepin
Ramsey
Minneapolis
St. Paul
Carver
Scott
Dakota
Number of People in Zip Code Reporting Speaking a Primary Language Other than English in the Home Per 1000 Population
No Data
0.00 - 22.8
22.9 - 48.3
48.4 - 85.7
85.8 - 139.7
139.8 - 229.6
229.7 - 484.5
Number of Malaria Cases by Zip Code 2010-2014
1 - 2
3 - 5
6 - 8
9 - 14
15 - 18
N
0 2.5 5 10 Miles

D

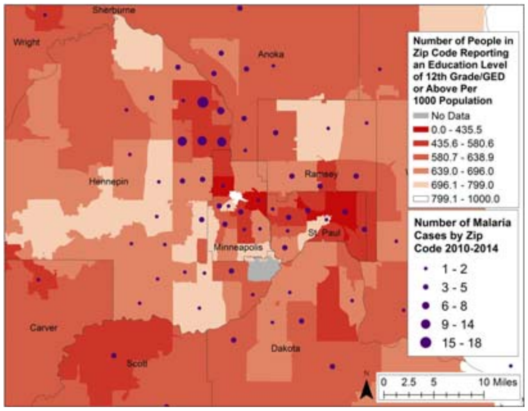
Sherburne
Wright
Anoka
Hennepin
Ramsey
Minneapolis
St. Paul
Carver
Scott
Dakota
Number of People in Zip Code Reporting an Education Level of 12th Grade/GED or Above Per 1000 Population
No Data
0.0 - 435.5
435.6 - 580.6
580.7 - 638.9
639.0 - 696.0
696.1 - 799.0
799.1 - 1000.0
Number of Malaria Cases by Zip Code 2010-2014
1 - 2
3 - 5
6 - 8
9 - 14
15 - 18
N
0 2.5 5 10 Miles

E

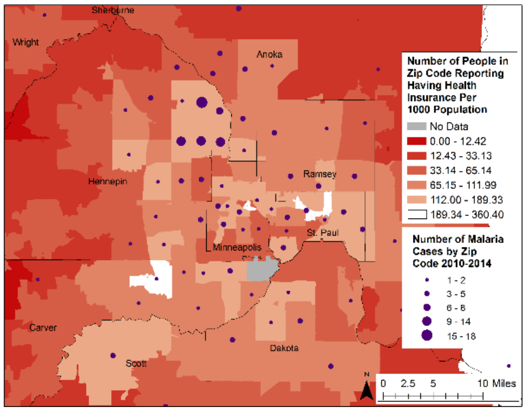
Sherburne
Wright
Anoka
Hennepin
Ramsey
Minneapolis
St. Paul
Carver
Scott
Dakota
Number of People in Zip Code Reporting Having Health Insurance Per 1000 Population
No Data
0.00 - 12.42
12.43 - 33.13
33.14 - 65.14
65.15 - 111.99
112.00 - 189.33
189.34 - 360.40
Number of Malaria Cases by Zip Code 2010-2014
1 - 2
3 - 5
6 - 8
9 - 14
15 - 18
N
0 2.5 5 10 Miles

F

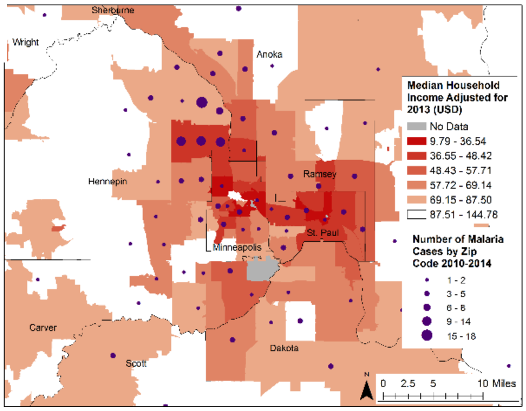
Sherburne
Wright
Anoka
Hennepin
Ramsey
Minneapolis
St. Paul
Carver
Scott
Dakota
Median Household Income Adjusted for 2013 (USD)
No Data
9.79 - 36.54
36.55 - 48.42
48.43 - 57.71
57.72 - 69.14
69.15 - 87.50
87.51 - 144.78
Number of Malaria Cases by Zip Code 2010-2014
1 - 2
3 - 5
6 - 8
9 - 14
15 - 18
N
0 2.5 5 10 Miles

G

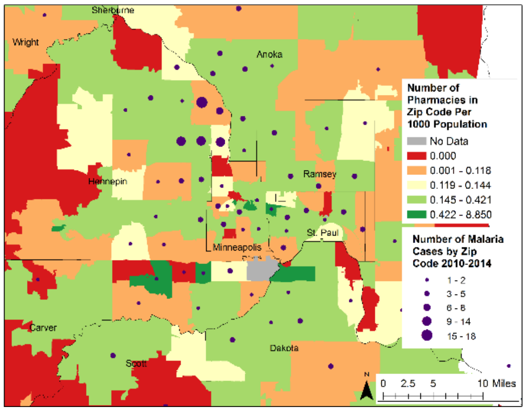
Sherburne
Wright
Anoka
Hennepin
Ramsey
Minneapolis
St. Paul
Carver
Scott
Dakota
Number of Pharmacies in Zip Code Per 1000 Population
No Data
0.000
0.001 - 0.118
0.119 - 0.144
0.145 - 0.421
0.422 - 8.850
Number of Malaria Cases by Zip Code 2010-2014
1 - 2
3 - 5
6 - 8
9 - 14
15 - 18
N
0 2.5 5 10 Miles

H

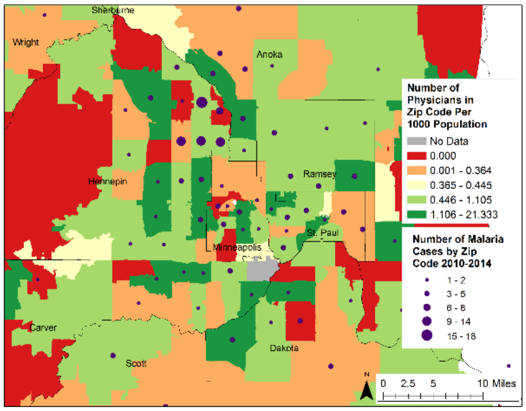
Sherburne
Wright
Anoka
Hennepin
Ramsey
Minneapolis
St. Paul
Carver
Scott
Dakota
Number of Physicians in Zip Code Per 1000 Population
No Data
0.000
0.001 - 0.364
0.365 - 0.445
0.446 - 1.105
1.106 - 21.333
Number of Malaria Cases by Zip Code 2010-2014
1 - 2
3 - 5
6 - 8
9 - 14
15 - 18
N
0 2.5 5 10 Miles

Supplemental Figure 3. Sensitivity analysis of PRIDD Method: Comparison of Reported to Predicted Imported Malaria Cases for Selected Minnesota ZIP Code Tabulation Areas 2010-2014. Reported cases by ZCTA are indicated by gray bars, while predicted case values are indicated by the blue line, with upper and lower 95% confidence intervals indicated by gray lines above and below the blue predicted values, respectively. ZCTAs were included (n=120) if the predicted value, lower, and upper bounds were all at least 1 case.

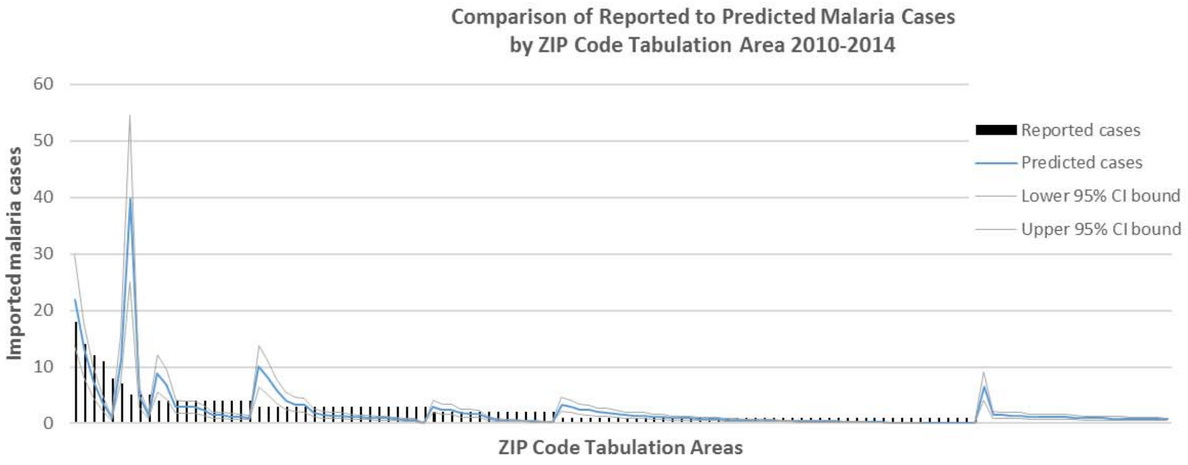

Supplemental Figure 4. Side by side comparison of actual and predicted imported malaria cases in Minnesota aggregated to the ZCTA-level for 2010-2014. Supplemental Figure 4A depicts reported malaria cases in the Minneapolis-St. Paul area for 2010-2014 by ZCTA. Supplemental Figure 4B depicts predicted malaria cases in the Minneapolis-St. Paul area for 2010-2014 by ZCTA.

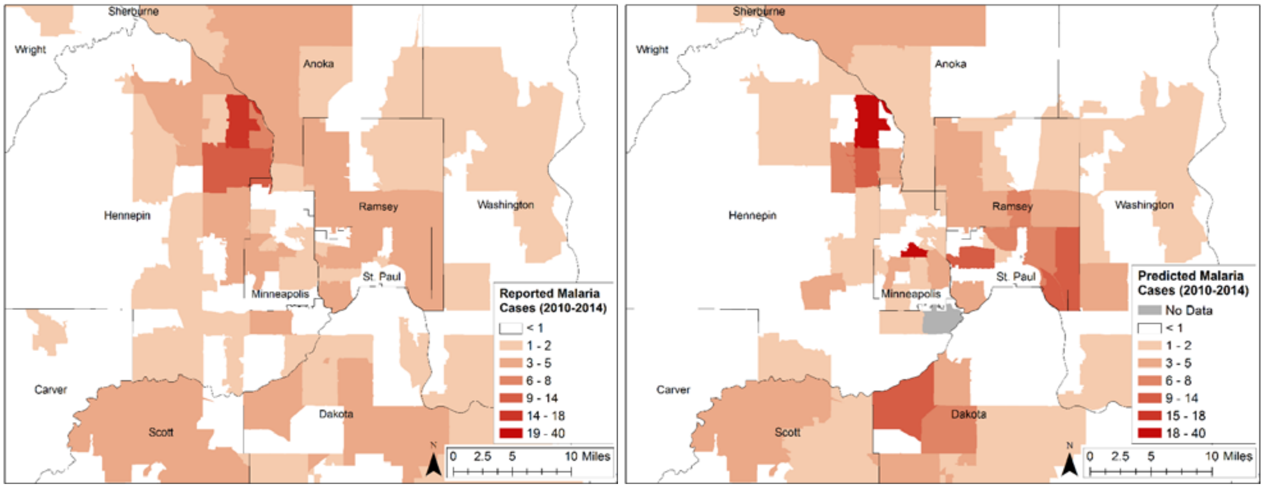

1A **1B**
